# Folding and Unfolding of Exogenous G-Rich Oligonucleotides in Live Cells by Fluorescence Lifetime Imaging Microscopy of *o*-BMVC Fluorescent Probe

**DOI:** 10.3390/molecules27010140

**Published:** 2021-12-27

**Authors:** Ting-Yuan Tseng, Chiung-Lin Wang, Wei-Chun Huang, Ta-Chau Chang

**Affiliations:** Institute of Atomic and Molecular Sciences, Academia Sinica, Taipei 10617, Taiwan; q102081@gmail.com (C.-L.W.); chun5200@gate.sinica.edu.tw (W.-C.H.)

**Keywords:** fluorescence lifetime imaging microscopy, G4 dynamics in live cells, *o*-BMVC fluorescent probe

## Abstract

Guanine-rich oligonucleotides (GROs) can self-associate to form G-quadruplex (G4) structures that have been extensively studied in vitro. To translate the G4 study from in vitro to in live cells, here fluorescence lifetime imaging microscopy (FLIM) of an *o*-BMVC fluorescent probe is applied to detect G4 structures and to study G4 dynamics in CL1-0 live cells. FLIM images of exogenous GROs show that the exogenous parallel G4 structures that are characterized by the *o*-BMVC decay times (≥2.4 ns) are detected in the lysosomes of live cells in large quantities, but the exogenous nonparallel G4 structures are hardly detected in the cytoplasm of live cells. In addition, similar results are also observed for the incubation of their single-stranded GROs. In the study of G4 formation by ssHT23 and hairpin WT22, the analyzed binary image can be used to detect very small increases in the number of *o*-BMVC foci (decay time ≥ 2.4 ns) in the cytoplasm of live cells. However, exogenous ssCMA can form parallel G4 structures that are able to be detected in the lysosomes of live CL1-0 cells in large quantities. Moreover, the photon counts of the *o*-BMVC signals (decay time ≥ 2.4 ns) that are measured in the FLIM images are used to reveal the transition of the G4 formation of ssCMA and to estimate the unfolding rate of CMA G4s with the addition of anti-CMA into live cells for the first time. Hence, FLIM images of *o*-BMVC fluorescence hold great promise for the study of G4 dynamics in live cells.

## 1. Introduction

A large number of guanine (G)-rich sequences are found in the human genome [1,2,3]. Such sequences may form G-quadruplex (G4) structures by stacking the G-quartets via Hoogsteen hydrogen bonding of four cyclic guanines under physiological conditions [4,5,6]. The major challenge is to verify the existence of G4 formation in cells [7]. Thus, a number of methods have been developed to detect and visualize G4 structures in cells [8,9,10,11]. Recently, fluorescence lifetime imaging microscopy (FLIM) was applied to a G4 fluorescent probe to detect and visualize the G4 structures in cells [12,13,14]. This method was also developed as a novel method to diagnose cancerous thyroid nodules [15]. Here, a fluorescent probe of the 3,6-bis(1-methyl-2-vinylpyridinium) carbazole diiodide (*o*-BMVC) is useful to distinguish G4 from other DNA structures [9]. The *o*-BMVC molecule has a better binding affinity to telomeric G4 structures than duplex DNA by approximately two orders of magnitude and a binding effect that is even better than that of single-stranded DNA (ssDNA) as well as longer fluorescent decay times upon interaction with 20 different G4 structures (≥2.4 ns) than 10 different non-G4 structures, including ssDNA and duplex DNA (<1.6 ns) [9,12]. Here, we introduce the potential uses of FLIM images of *o*-BMVC fluorescence for the study of G4 in live cells.

It is known that some G-rich oligonucleotides (GROs), such as PU27 [16], AS1411 [17], and HT24 [18], have been studied as potential therapeutic candidates for cancer treatment. Previously, we examined the cellular uptake of these exogenous GROs and visualized their intracellular localizations in live cells by means of confocal microscopy [19]. The Cy5-labeled GROs showed that the Cy5-PU22 and Cy5-AS1411 G4s were retained and detected in the lysosomes, while the Cy5-HT23 and Cy5-TBA G4s were probably distorted or degraded and distributed in the cytoplasm of live CL1-0. Notably, the circular dichroism (CD) spectra of PU22 and AS1411 showed a positive band near 265 nm and a negative band near 240 nm in K^+^ solution, which are both characteristics of parallel G4 structures. The CD spectra of HT23 and TBA showed a positive band near 290 nm in K^+^ solution, which is a major characteristic of non-parallel G4 structures. The parallel and non-parallel forms of the G4 structures were previously defined by the relative orientations of the strands and the glycosidic conformations of the guanines [20]. For simplicity, the parallel form has four strands that are oriented in the same direction, while the non-parallel G4 form has either one or two strands that are oriented in the opposite direction. In addition, telomeric GROs were shown to be able to convert from non-parallel forms to parallel forms in a crowded 2M polyethylene glycol solution [21]. However, little is known about which types of G4 structures are favorably formed in live cells.

Given that the occurrence of G4 DNA is a hallmark of the cancer genome [22,23], G4 ligands may act as potential anticancer agents to inhibit the growth of cancer cells. Notably, biological functions of DNA depend on their structure and stability as well as their conformational dynamics. Thus, the details of the G4 structures and dynamics in live cells are critical not only for studying the biological function but also for designing the G4 ligands. Currently, the translation of G4 study from in vitro to in live cells is a key topic in G4 research. In this work, we first used FLIM to measure the fluorescence lifetimes of the 3,6-bis(1-methyl-2-vinylpyridinium) carbazole diiodide (*o*-BMVC) upon binding to these exogenous G4 structures in live cells to confirm whether the G4 structures can be maintained in their parallel form but not in their non-parallel form in live cells. To elucidate whether this difference is determined by structure or sequence, we further examined the possible G4 formation from exogenous single-stranded GROs in live cells. In addition, a G-rich sequence from the *WNT1* promoter region, WT22, which forms a hairpin structure in tris buffer, can adopt a non-parallel G4 structure in K^+^ solution [24]. Given that the signals of *o*-BMVC are characterized by their decay time (≥2.4 ns) and are hardly detected in live cells upon binding to non-parallel G4s, time-gated FLIM images were analyzed using the Otsu threshold method [25] to decrease the possible counting errors that might occur as a result of human eye detection and to unambiguously quantify the number of *o*-BMVC foci (decay time ≥ 2.4 ns) in live cells [12]. The quantitative measurement of the number of *o*-BMVC foci allowed us to reveal G4 formation from exogenous ssGROs in live cells under different conditions.

Moreover, we use FLIM images of *o*-BMVC to examine the folding and unfolding behaviors of exogenous CMA G4 structures in live cells. The CMA is a G-rich sequence from the 5′-end of the c-MYC promoter NHE III_1_, which can form a parallel G4 structure in K^+^ solution [26]. Given that the *o*-BMVC signals are characterized by the decay time (≥2.4 ns) are strong in the lysosomes of live CL1-0 cells upon binding to parallel G4 structure, the photon counts of the *o*-BMVC fluorescence in the FLIM images were measured to study the G4 formation of exogenous ssCMA and to measure the unfolding time of exogenous CMA G4 structures after the addition of its antisense DNA in live CL1-0 cells. Table 1 lists the GROs that were studied in this work together with the center peaks of the *o*-BMVC decay times that were detected in solution as well as in live cells upon binding to these G4 structures.

## 2. Results

### 2.1. FLIM Images of o-BMVC for Monitoring the Exogenous G4s in CL1-0 Live Cells

**Figure 1:**
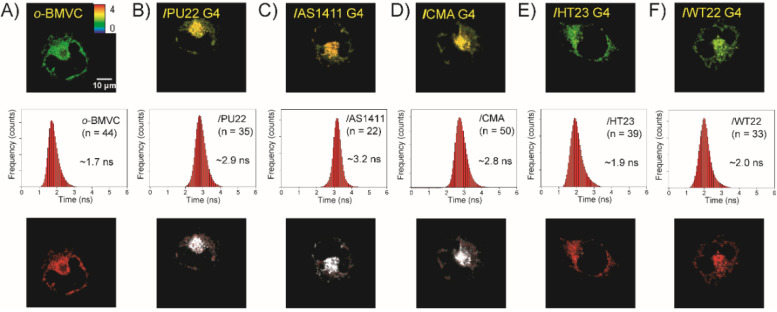
FLIM images of (**A**) 5 μM *o*-BMVC and its complexes with the G4 structures of 15 μM (**B**) PU22, (**C**) AS1411, (**D**) CMA, (**E**) HT23, and (**F**) WT22 when incubated with live CL1-0 cells for 2 h together with their histograms of the decay times of the *o*-BMVC fluorescence (n is the number of cells) and their time-gated FLIM images with the time threshold at 2.4 ns to separate the images into two colors: white (decay time ≥ 2.4 ns) and red (decay time < 2.4 ns), in live cells.

Figure 1A–F show the FLIM images of 5 μM *o*-BMVC and its mixtures with G4 structures belong to 15 μM PU22, AS1411, CMA, HT23, and WT22 that had been incubated with CL1-0 cancer cells for 2 h as well as the histograms of the decay time of the *o*-BMVC fluorescence in those mixtures and the time-gated FLIM images of those mixtures, respectively. The time-gated FLIM images were obtained by separating the FLIM images into two colors: white (≥2.4 ns) and red (<2.4 ns). The results show that the parallel G4 structures of PU22, AS1411, and CMA are largely detected in the lysosomes, while the non-parallel G4 structures of HT23 and WT22 are rarely detected in the lysosomes of live CL1-0 cells. The FLIM findings are consistent with the previous FRET results [19]. Something that is of importance is that the decay times of the *o*-BMVC fluorescence upon binding to these parallel G4 structures that were detected in the lysosomes of the live cells are almost identical to those detected in solution [12], implying that the parallel G4 structures are retained in live cells in the same way that they are in K^+^ solution. For the non-parallel G4 structures of HT23 and WT22, the fact that shorter decay times for the *o*-BMVC fluorescence were detected in the cytoplasm of live cells than those that were detected in solution suggests that the non-parallel G4 structures are either distorted or degraded in the lysosomes, resulting in the *o*-BMVC being released into the cytoplasm of the live CL1-0 cells.

### 2.2. FLIM Images of o-BMVC for Monitoring the Exogenous Single-Stranded HT23 in CL1-0 Live Cells

**Figure 2:**
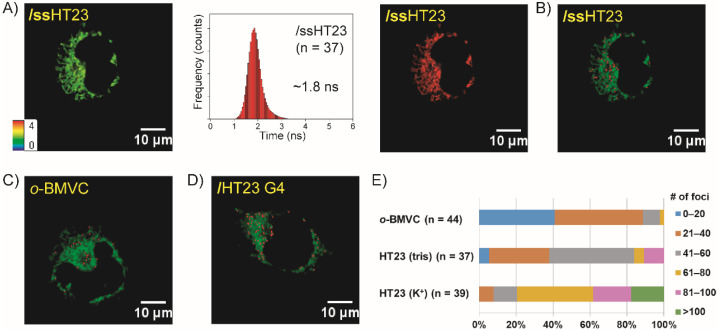
Imaging study of exogenous of HT23. FLIM images of (**A**) a mixture of 5 μM *o*-BMVC and 15 μM ssHT23 incubated with a live CL1-0 cell (**left**) and a histogram of the decay times of the *o*-BMVC fluorescence (**middle**) together with its time-gated image for 2 h (**right**). (**B**) Using the Otsu threshold method to analyze the time-gated FLIM image (**A**, **right**), the analyzed binary images were separated into two colors: red (decay time ≥ 2.4 ns) and green (decay time < 2.4 ns). The red spots were determind to be the *o*-BMVC foci. For comparison, the analyzed binary images of (**C**) *o*-BMVC and (**D**) the mixture of *o*-BMVC and HT23 G4s incubated with live CL1-0 cells for 2 h. (**E**) Quantitative analysis of the number of *o*-BMVC foci in these analyzed binary images.

To elucidate whether this difference is determined by structure or sequence, we further examined the possible G4 formation of exogenous single-stranded HT23 (ssHT23) in live CL1-0 cells. Figure 2A shows a FLIM image of a mixture of 5 μM *o*-BMVC and 15 μM ssHT23 incubated with CL1-0 cells for 2 h (left) together with a histogram of the decay time of the *o*-BMVC fluorescence (central) and its time-gated image (right). The time-gated image also shows that the *o*-BMVC signals are hardly detected in live cells upon binding to HT23 G4s (decay time ≥ 2.4 ns) (Figure 2A, right). The time-gated image can be further analyzed using the Otsu threshold method [25] to find an optimal threshold for *o*-BMVC foci detection (decay time ≥ 2.4 ns). The analyzed binary image provides method through which the number of *o*-BMVC foci (the red spots in Figure 2B) can be quantitatively measured in live cells. To verify whether the *o*-BMVC foci detection is due to the G4 formation from exogenous ssHT23, Figure 2C shows the analyzed binary image of *o*-BMVC that has been incubated with CL1-0 cells for 2 h. In addition, Figure 2D shows the analyzed binary image of HT23 G4s incubated with CL1-0 cells for 2 h. Quantitative measurements of the number of *o*-BMVC foci show that the number of *o*-BMVC foci that was detected for HT23 G4 incubation is more than that detected for ssHT23 incubation as well as in the absence of exogenous HT23 in live CL1-0 cells (Figure 2E).

### 2.3. FLIM Images of o-BMVC for Monitoring the Exogenous WT22 in CL1-0 Live Cells

**Figure 3:**
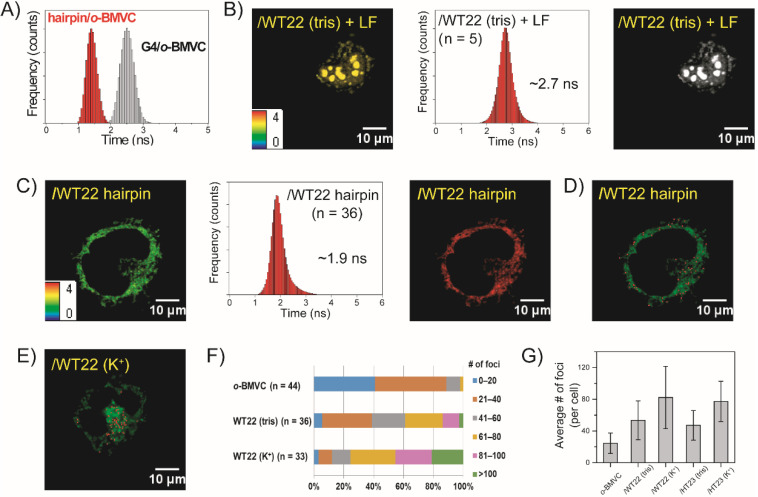
Imaging study of conformational change in exogenous WT22 from a hairpin structure to a G4 structure in live CL1-0 cells. (**A**) The histograms of the fluorescence decay time of *o*-BMVC upon interaction with WT22 in tris-buffer and in 150 mM K^+^ solution. (**B**) Lipofectamine was used to deliver a mixture of 1 μM *o*-BMVC and 3 μM hairpin WT22 into the nucleus of live CL1-0 cells overnight; the FLIM image (**left**) and histogram of the decay times of *o*-BMVC fluorescence (**middle**) together with its time-gated image are presented in the pseudocolors of white (decay time ≥2.4 ns), which resulted from the interaction with the G4 structures, and red (decay time < 2.4 ns), which represents intereactions with others (**right**). (**C**) FLIM image of a mixture of 5 μM *o*-BMVC and 15 μM hairpin WT22 incubated with live CL1-0 cells (**left**) and a histogram of the decay times of the *o*-BMVC fluorescence (**middle**) together with its time-gated image for 2 h (**right**). (**D**) Using the Otsu threshold method to analyze the time-gated FLIM image, the analyzed binary images were separated into two colors: red (decay time ≥ 2.4 ns) and green (decay time < 2.4 ns). (**E**) The analyzed binary images of WT22 G4s incubated with live CL1-0 cells for 2 h. (**F**) Quantitative analysis of the number of *o*-BMVC foci and (**G**) the average number of *o*-BMVC foci per cell and its mixtures with hairpin WT22 and WT22 G4 together with ssHT23 and HT23 G4 in live CL1-0 cells.

Recently, a conformational transition in WT22 from a hairpin structure to a non-parallel G4 structure was detected after the addition of 150 mM K^+^ in solution [24]. Figure 3A shows a histogram of the decay times of *o*-BMVC fluorescence upon interaction with the hairpin and G4 structures of WT22 in solution without and with the addition of K^+^, respectively. Here, we first used lipofectamine to deliver 1 μM *o*-BMVC mixed with 3 μM WT22 hairpin into the nucleus of live CL1-0 cells overnight. Figure 3B shows the FLIM image (left) together with a histogram of the decay time of the *o*-BMVC fluorescence (central) as well as its time-gated FLIM image (right). The time-gated image shows a number of large fluorescent spots that are characterized by the decay times (≥2.4 ns) in the nucleus, indicating that the conformational change in WT22 from a hairpin structure to a G4 structure is detected in the nucleus of live cells.

Figure 3C shows a FLIM image of a mixture of 5 μM *o*-BMVC and 15 μM hairpin WT22 incubated with live CL1-0 cells for 2 h (left) together with a histogram of the decay time of the *o*-BMVC fluorescence (central) and its time-gated FLIM image (right). The time-gated image shows that the *o*-BMVC signals with the decay time ≥2.4 ns are hardly detected in the live cells (Figure 3C, right). Notably, WT22 forms a mainly non-parallel G4 structure in K^+^ solution. Using the Otsu threshold method [25], the analyzed binary image allows us to measure the number of *o*-BMVC foci (the red spots in Figure 3D) in live cells. In addition, Figure 3E shows the analyzed binary image of the WT22 G4s that had been incubated with CL1-0 cells for 2 h. Again, quantitative measurements of the number of *o*-BMVC foci show that the number of *o*-BMVC foci that was detected for WT22 G4 incubation is more than that detected for hairpin WT22 incubation and more than that detected for the absence of exogenous GRO in the live cells (Figure 3F). In addition, the average number of *o*-BMVC foci that was detected in the analyzed binary images of the exogenous HT23 and WT22 in live cells are summarized in Figure 3G. Compared to the much more parallel G4 formation resulting from the exogenous GROs, only a very small portion of the exogenous hairpin WT22 and ssHT23 can form G4 structures in live cells.

### 2.4. Folding of Exogenous Single-Stranded CMA into G4 Structures in CL1-0 Live Cells

**Figure 4:**
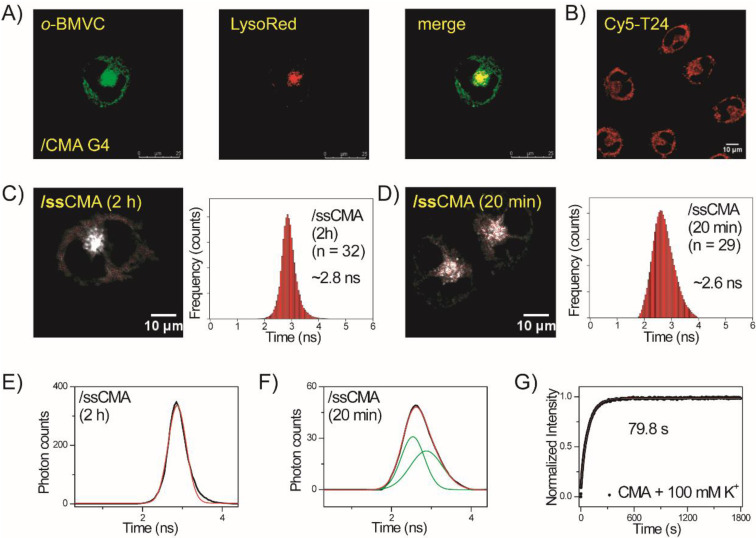
Imaging study of G4 folding of exogenous ssCMA in live cells. (**A**) Confocal images of a mixture of *o*-BMVC and CMA G4 structures incubated with a CL1-0 cancer cell for 2 h (**left**) and that was then stained with LysoTracker red (**middle**) together with their merges (**right**). (**B**) Confocal image of Cy5-T24 incubated with CL1-0 cancer cells for 2 h. The time-gated FLIM images of a mixture of 5 μM *o*-BMVC and 15 μM ssCMA incubated with live CL1-0 cells for (**C**) 2 h and (**D**) 20 min to study G4 formation together with a histogram of the *o*-BMVC decay times. (**E**) A curve fitting by a single band at 2.87 ns for the incubation of ssCMA for 2 h and (**F**) a curve fitting by two bands at 2.53 ns and 2.87 ns for the incubation of ssCMA for 20 min. (**G**) The CD signals at 265 nm are a function of time to study G4 formation of ssCMA after the addition of 100 mM K^+^ in solution.

To confirm that the CMA G4s are able to be detected in the lysosomes of live CL1-0 cells, a confocal image of a mixture of 5 μM *o*-BMVC and 15 μM of CMA G4s incubated with CL1-0 cancer cells for 2 h is shown in Figure 4A (left). LysoTracker red was used to determine the intracellular location of CMA in the live CL1-0 cells (Figure 4A, central). The merge between the LysoTracker red and the *o*-BMVC (Figure 4A, right) confirms that the lysosomes are the major location of CMA G4s in live CL1-0 cells. In addition, Figure 4B shows a confocal image of Cy5-labeled ssDNA (Cy5-T24) incubated with live CL1-0 cells for 2 h, implying that ssDNA can escape from the lysosomes.

Given that the ssDNA can escape from the lysosomes, it is necessary to examine whether ssCMA can fold into G4 structures before its escape from the lysosomes. Figure 4C shows the time-gated FLIM image of a mixture of 5 μM *o*-BMVC and 15 μM ssCMA incubated with live CL1-0 cells for 2 h (left) together with a histogram of the *o*-BMVC fluorescence decay time (right). Given that the detection of *o*-BMVC signals (decay time ≥ 2.4 ns) in the lysosomes not only depends on the folding of ssCMA but also on the uptake of ssCMA into the lysosomes, Figure 4D shows a time-gated FLIM image (left) and a histogram (right) of a mixture of *o*-BMVC and ssCMA incubated with live CL1-0 cells for 20 min. The detection of strong *o*-BMVC signals (decay time ≥ 2.4 ns) in the lysosomes suggests that the G4 formation from ssCMA takes place within 20 min. Of interest is that the histogram of the *o*-BMVC decay time shows a slight shift in the center from about 2.6 ns for 20 min of ssCMA incubation to about 2.85 ns for 2 h of ssCMA incubation with live CL1-0 cells. The former one fitted by two bands with the center at 2.53 and 2.87 ns shows very good results (Figure 4F), while the other one that is fitted by a single band with its center at 2.87 ns shows slight deviation near 3.5 ns (Figure 4E). In addition, CD spectroscopy was used to determine the folding rate of ssCMA after the addition of 100 mM K^+^. The arising time of CD signal at 265 nm is fitted to a single exponential function with a time constant of about 80 sec (Figure 4G).

### 2.5. Unfolding of Exogenous CMA G4 Structure by Its Anti-CMA in CL1-0 Live Cells

**Figure 5:**
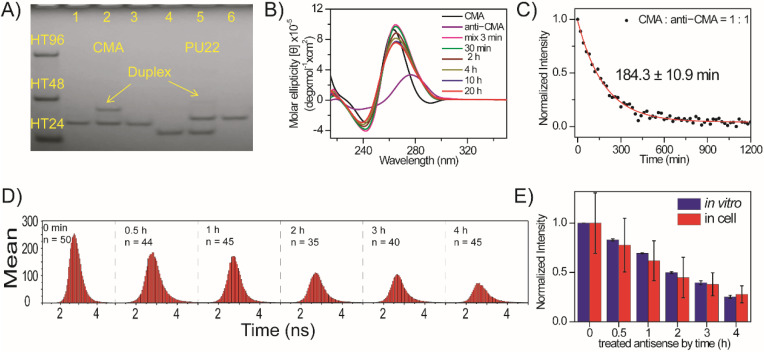
Imaging study of folding and unfolding of exogenous CMA in live CL1-0 cells. (**A**) Polyacrylamide gel electrophoresis assays of marker bands of HT24 (T_2_AG3)_4_, HT48 (T_2_AG_3_)_8_, and HT96 (T_2_AG_3_)_16_ and CMA G4s (lane 1), CMA G4s after the addition of anti-CMA overnight (lane 2), anti-CMA (lane 3), PU22 G4 (lane 4), PU22 G4 after addition of anti-PU22 overnight (lane 5), and anti-PU22 (lane 6). (**B**) CD spectra of CMA and anti-CMA together with their mixture at different times in 150 mM K^+^ solution. (**C**) The decrease in the CD signals of CMA G4s at 265 nm is fitted to a single exponential function with a time constant of 184 ± 11 min. (**D**) The histograms of the average photon counts vs. the decay times of the *o*-BMVC signals in each cell after the addition of anti-CMA at different times. (**E**) The plots of the average photon counts of *o*-BMVC signals per cell and the CD signals of CMA at 265 nm in solution after the addition of anti-CMA at different times, respectively.

The use of an antisense sequence was previously applied to unfold human telomeric G4 structures in vitro [27,28] and in vivo [29]. In the presence of antisense DNA, some of the unfolded species that formed as a result of thermal motion can be trapped as a stable duplex. The portions that shift from G4 to duplex structures depend on G-rich sequences. Here, PAGE assays show that the use of 30 μM anti-CMA to unfold 30 μM CMA G4 structures is more distinct than the use of anti-PU22 to unfold PU22 G4 structures (Figure 5A). In addition, Figure 5B shows the CD spectra of 20 µM CMA and 20 µM anti-CMA together with their mixture at 3 min, 30 min, 2 h, 4 h, 10 h, and 20 h in 150 mM K^+^ solution. The decrease in the CD signal at 265 nm of CMA G4 structures after the addition of anti-CMA is fitted to a single exponential function with a time constant of about 185 min (Figure 5C).

It is important to examine whether the use of anti-CAM can unfold the exogenous CMA G4 structures in live cells. After mixture of 5 μM *o*-BMVC and 15 μM CMA G4s was incubated with live CL1-0 cells for 2 h, the sample was washed with PBS twice followed by the addition of 15 μM anti-CMA. Figure 5D shows the histograms of the average photon counts of the *o*-BMVC fluorescence per cell before and after the addition of anti-CMA at 0.5 h, 1 h, 2 h, 3 h, and 4 h. Figure 5E shows the plots of the average photon counts of *o*-BMVC fluorescence per cell together with the CD signals of CMA at 265 nm before and after the addition of anti-CMA at 0.5 h, 1 h, 2 h, 3 h, and 4 h. Notably, the deviation in the photon counts of the *o*-BMVC fluorescence that were measured in live cells was much larger than the deviation in the CD intensity of the G4 structures that were measured in solution. At present, it is difficult to achieve a reliable measurement of the photon count for the *o*-BMVC fluorescence after the addition of anti-CMA for longer amounts of time (>4 h).

We further measured the uptake ratio of *o*-BMVC binding to the CMA G4s that remained in the lysosomes of the live CL1-0 cells after the sample had been washed in PBS for 4 h. The results show that approximately 80% of the CMA G4s remained in the lysosomes of the live cells after a 4 h delay time (Figure S1). Although such a decrease can contribute to the unfolding rate in live cells, the results suggest that the unfolding rate of the CMA G4 structures when using anti-CMA in live cells is the same time scale as it is in solution. To the best of our knowledge, this is the first report to study the unfolding rate of the G4 structure in live cells.

## 3. Discussion

The importance of G4 structures has been associated with genome instability, genetic diseases, and cancer progression [7,30,31]. Accumulating studies in G4 detection support the existence of G4 structures within cells [8,9,10,11,12,13,14]. However, it is not clear which types of G4 structures are favorably formed in live cells. Generally, CD spectroscopy provides a simple tool to distinguish parallel from non-parallel G4 structures in vitro. Here, FLIM images of the *o*-BMVC fluorescence were used as a quick method to recognize parallel G4 structures from exogenous GROs in live CL1-0 cells. In addition to PU22, AS1411, and CMA, a preliminary test of several G-rich sequences, such as T4T-1 (5′-G_3_TG_3_ATTAG_3_TG_3_), TTT (5′-G_3_TG_3_TG_3_TG_3_), T4T-1-FN (5′-TGAG_3_TG_2_ATTAG_3_TG_3_TAA), and TTT-FN (5′-TGAG_3_TG_3_TG_3_TG_3_TAA), showed a positive CD band at 265 nm accompanied by a negative band at 240 nm as typical signals of parallel G4 structures in 100 mM K^+^ solution [32]. The images of these ssGROs incubated with CL1-0 live cells all show a large accumulation of *o*-BMVC signals in the lysosomes (Figure S2). Notably, T4T-1 and TTT are predominated by dimeric G4 structures, while T4T-1-FN and TTT-FN are predominated by monomeric G4 structures in 100 mM K^+^ solution. Moreover, RNA HT23 (5′-UAG_3_(U_2_AG_3_)_2_) also shows a positive CD band at 265 nm and a negative band near 240 nm in 150 mM K^+^ solution, and RNA HT23 incubation with CL1-0 live cells also shows the large accumulation of *o*-BMVC signals in the lysosomes, confirming the formation of a parallel G4 structure. Here, the accumulation of parallel G4 structures from the exogenous ssGROs that were revealed by the *o*-BMVC signals (decay time ≥ 2.4 ns) in the lysosomes suggests that this is a promising method that can be used to recognize the parallel G4 structures in live CL1-0 cells.

The FLIM images showed that the incubation of parallel G4 structures can be retained in the lysosomes, while the incubation of non-parallel G4 structures cannot be detected in the lysosomes of live CL1-0 cells. In addition, similar results are also observed during the incubation of their ssGROs. Considering that some ssGROs can quickly form parallel G4 structures, one possibility is that other ssGROs can quickly form non-parallel G4 structures in the lysosomes. If true, then the lysosomes play a key role in determining the retention of the parallel form as well as in the degradation of the non-parallel form of G4 structures. Notably, one of the important roles of lysosomes is to degrade intracellular and exogenous macromolecules, such as DNA fragments, into building blocks for further utilization [33,34]. At present, it is not clear why the parallel G4 structures are retained and the non-parallel G4 structures are distorted or degraded in the lysosomes.

However, the analyzed binary images show very small increases in the number of *o*-BMVC foci in the cytoplasm due to the incubation of the HT23 and WT22 G4s with live CL1-0 cells. It is known that G-rich sequences can adopt various G4 structures; for example, there are at least five different monomeric G4 structures that are formed by slightly different human telomeric sequences, including four non-parallel and one parallel G4 structures [35,36,37,38,39]. In addition, different G4 structures from a G-rich sequence could be detected in in vitro and in live cells because of the different environments. Miyoshi and Sugimoto [40] recognized the difference between solution and intracellular conditions, and they used polyethylene glycol (PEG) to mimic the cellular crowding environment and found that 2M PEG can not only induce conformation change but can also increase the stability of the G4 structure. Heddi and Phan [21] reported that the use of polyethylene glycol (PEG) as a co-solvent can convert four different non-parallel G4 structures to parallel G4 structures due to water depletion. Moreover, we took advantage of this property of PEG to design a hybrid ligand, BMVC–8C3O, by means of the covalent attachment of a PEG unit to the G4 ligand, BMVC [41]. Indeed, BMVC*–*8C3O can induce structural change from different non-parallel G4 structures to their parallel G4 structures. It was suggested that these conformational changes in human telomeres from non-parallel to parallel G4 structures is induced by dehydration. Here, the detection of small increases in the *o*-BMVC foci from the incubation of HT23 with live cells may be due to parallel G4 formation. Although we suggest that the non-parallel G4 structures are unlikely to be favored in the cytoplasm of CL1-0 live cells, we are not able to eliminate the possible existence of non-parallel G4 structures in the cytoplasm of CL1-0 live cells.

The strong *o*-BMVC signals that are detected in the lysosomes provide useful information for the study of folding and unfolding behaviors of the G4 structures of CMA in live cells. For example, an imaging study of G4 formation from ssCMA shows the change from two binding modes to one binding mode as a function of time in live cells. To our knowledge, this is the first time that such a finding is reported in live cells. Here, we propose that band 1, which is characterized by the ~2.54 ns at the band center, is the intermediate state of band 2, which is characterized by the ~2.85 ns at the band center. It is likely that band 1 is the result of *o*-BMVC binding to monomeric G4s and that band 2 is due to the sandwiching of *o*-BMVC between two G4s. This finding suggests that the stacking of two parallel G4 structures is feasible in live cells.

The histograms of the *o*-BMVC decay time for the unfolding of the CMA G4 structures also show a shift from ~2.8 ns before the addition of anti-CMA to ~2.65 ns after the addition of anti-CMA for 4 h. The difference between these two distributions is a slight increase in photon counts at ~2 ns and a significant decrease in photon counts at ~2.8 ns (Figure S3). This difference is due to the unfolding of the G4 structures due to the increased *o*-BMVC that is released from the G4 binding, which increases photon counts at shorter decay times near the 2.0 ns center, which is associated with a decrease in the photon counts at longer decay times near the 2.8 ns center. This finding further supports the previous hypothesis of two binding modes.

In summary, we demonstrate that FLIM images of *o*-BMVC fluorescence provide a promising tool that can be used to detect and visualize G4 structures and to study G4 dynamics in live cells. It is important to note that ssGROs are favored for the formation of parallel G4 structures but that they are not favored to form non-parallel G4 structures in the lysosomes of live cells, which provide a tool to distinguish parallel from nonparallel G4 structures in live cells. However, such differences due to the lysosomes regulating parallel and nonparallel G4 structures deserve further study in order to disclose the mechanism that is involved. In addition, the analyzed binary images can be used to detect a very small increase in the *o*-BMVC foci resulting from the incubation of exogenous HT23 and WT22 in live cells. Moreover, the photon counts of the *o*-BMVC signals (decay time ≥ 2.4 ns) can be measured to reveal folding and unfolding behaviors of the G4 structures in live cells. To our knowledge, this is the first time that G4 formation in CMA as the result of conformational change as a function of time was detected in live cells as well as the first time that the time scale for the unfolding time of CMA G4 structures through the use of anti-CMA was measured in live cells as well as in solution. Furthermore, lysosomal dysfunction can lead to the accumulation of un-degraded material, which may result in lysosomal storage diseases. Thus, this work may open a new direction for the exploration of possible roles of lysosomes in regulating GROs for therapeutic applications.

## 4. Materials and Methods

### 4.1. Chemical Properties of o-BMVC Molecule

The synthesis of *o*-BMVC can be found elsewhere [9]. This molecule barely fluoresces in buffer but strongly fluoresces upon interaction with G4 structures by nearly two orders of magnitude.

### 4.2. Fluorescence Lifetime Imaging Microscopy (FLIM)

The setup of the FLIM system consisted of a picosecond diode laser (laser power, 5 mW) with an emission wavelength of 470 nm (LDH470; PicoQuant, Berlin, Germany) and a ~70 ps pulse width for the excitation of o-BMVC under a scanning microscope (IX-71 and FV-300; Olympus, Tokyo, Japan). The fluorescent signal from the *o*-BMVC was collected using a 60 × NA = 1.42 oil-immersion objective (PlanApoN; Olympus, Tokyo, Japan) passing through a 550/88 nm bandpass filter (Semrock, Rochester, New York, NY, USA), followed by detection using a SPAD (PD-100-CTC; Micro Photon Devices, Bolzano, Italy), the time resolution of which was was less then 50 ps FWHM. The fluorescence lifetime was recorded and analyzed using a time-correlated single-photon counting (TCSPC) module and software by mono-exponential curve fitting (PicoHarp 300, electrical time resolution was less then 25 ps and SymPhoTime v5.3.2; PicoQuant, Berlin, Germany). FLIM images were constructed from pixel-by-pixel lifetime information. Time-gated FLIM images had set time windows at 2.4 ns to separate the image into two colors: white (decay time ≥ 2.4 ns) and red (decay time < 2.4 ns). Live CL1-0 cells were incubated with 5 µM *o*-BMVC and its complexes with 15 µM GROs for 2 h. For the unfolded study, samples were washed twice with phosphate-buffered saline (PBS) followed by the addition of 15 µM anti-CMA.

### 4.3. Confocal Microscopy

CL1-0 cells were treated with 15/5 μM CMA/*o*-BMVC for 2 h and were co-stained with 20 nM LysoTracker red DND-99 (Invitrogen, Waltham, MA, USA) for 10 min. For the study of fluorophore-labeled ssDNA sequences, 15 μM Cy5-T24 were used to treat the CL1-0 cells for 2 h. Samples were washed twice with PBS and were visualized using a confocal microscope (Leica TCS SP8; Leica, Wetzlar, Germany).

### 4.4. Circular Dichroism (CD)

CD experiments were conducted using a spectropolarimeter (J-815, Jasco, Tokyo, Japan) with a bandwidth of 2 nm at a scan speed of 50 nm/min and at a step resolution of 0.2 nm over a spectral range of 210–350 nm. The sense DNA concentration of each sample was 20 μM dissolved in 10 mM Tris (pH 7.5), and a stock solution of 3 M KCl (Sigma-Aldrich, Burlington, MA, USA) was added to the DNA samples to attain a final K^+^ concentration. The observed signals were subtracted from the baseline. The melting curves were recorded at 265 nm or 290 nm from 20 to 95 °C, with a temperature ramping rate of 1 °C/min rate that was controlled with a Peltier thermal coupler chamber (PFD-425S/15, Jasco, Tokyo, Japan).

### 4.5. Polyacrylamide Gel Electrophoresis (PAGE)

PAGE was conducted using 16% polyacrylamide and 0.5× TBE gels in the presence of 20 mM K^+^. The sense and anti-sense DNA concentration of each sample was 30 μM. PAGE was conducted at 175 V for 225 min at 10 °C. Gels were then photographed under ultraviolet (UV) light at 254 nm using a digital camera.

### 4.6. Cell Cultures

CL1-0, a human lung carcinoma cancer cell line, was kindly provided by Prof. P. C. Yang (National Taiwan University) [42]. CL1-0 cells were cultured in RPMI1640 medium supplemented with 10% fetal bovine serum (FBS) and 1% antibiotics. Cell lines were cultured in 5% CO_2_ at 37 °C. The antibiotic concentration was 100 U/mL penicillin and streptomycin.

## Figures and Tables

**Table 1:** The GROs used in this work and the center peaks of the *o*-BMVC decay times upon binding to these G4 structures.

Name	Sequence	Fluorescent Decay Times	
		In Vitro	In Live Cells
PU22	TGAG_3_TG_4_AG_3_TG_4_AA	3.0	2.9
AS1411	(GGT)_4_TG(TGG)_4_	3.1	3.2
CMA	TAG_3_AG_3_TAG_3_AG_3_T	3.1	2.8
HT23	TAG_3_(T_2_AG_3_)_3_	2.8	-
WT22	G_3_CCACCG_3_CAG_5_CG_3_	2.5	2.7

## Supplementary Materials

The following supporting information can be downloaded, Figure S1: The plots of the average photon counts of *o*-BMVC fluorescence per cell, Figure S2: FLIM images, Figure S3: CD studies of CMA in vitro.

## Sample Availability

Samples of the compounds are available from the authors.
