# Supplementary material for: Folding and Unfolding of Exogenous G-Rich Oligonucleotides in Live Cells by Fluorescence Lifetime Imaging Microscopy of o-BMVC Fluorescent Probe
Source: Molecules. 2021 Dec 27;27(1):140. doi: 10.3390/molecules27010140 (PMC8747072; doi:10.3390/molecules27010140)
Supplement: Supplementary file 1 [file molecules-27-00140-s001.zip › molecules-1517675-supplementary.pdf]

Supporting information

Folding and unfolding of exogenous G-rich oligonucleotides in live cells by fluorescence lifetime imaging microscopy of *o*-BMVC fluorescent probe

Ting-Yuan Tseng*, Chiung-Lin Wang, Wei-Chun Huang and Ta-Chau Chang*

Institute of Atomic and Molecular Sciences, Academia Sinica, Taipei, Taiwan 10617

* Corresponding author: Ting-Yuan Tseng (email: homeotic@gmail.com) or Ta-Chau Chang (email: tcchang@pub.iams.sinica.edu.tw)

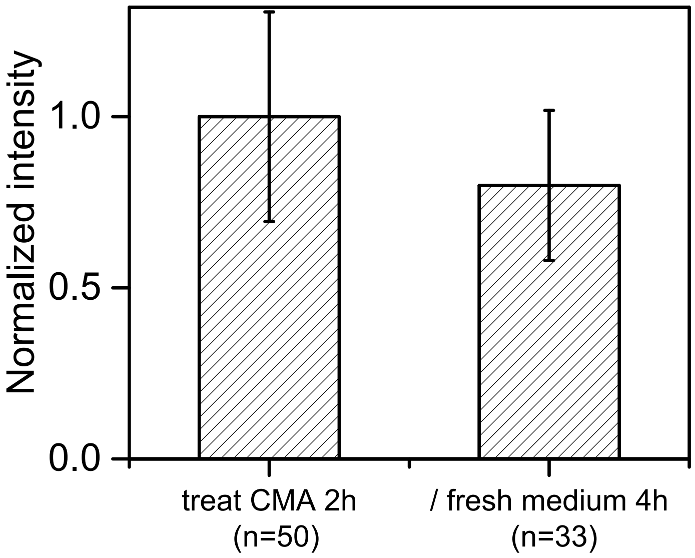

Figure S1. The plots of the average photon counts of *o*-BMVC fluorescence per cell. The ratio of the uptake of *o*-BMVC binding to CMA G4 structures remained in the lysosome of CL1-0 live cells after the sample removed CMA for 4 h.

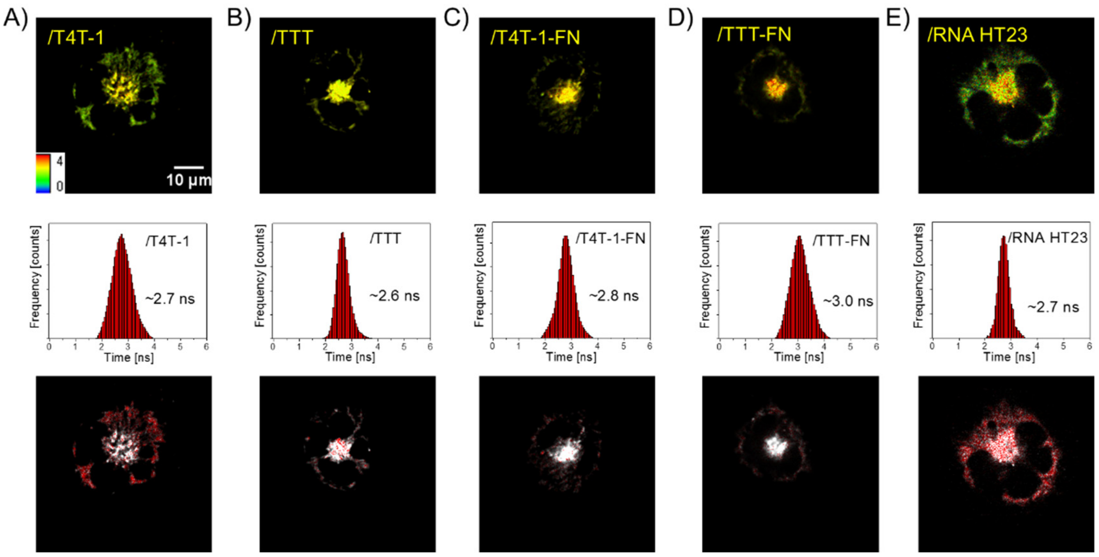

Figure S2. FLIM images of a mixture of 5 μM *o*-BMVC and 15 μM A) T4T-1, B) TTT, C) T4T-1-FN, D) TTT-FN, and E) RNA HT23 incubated with CL1-0 live cells for 2 h together with their histograms of the decay time of *o*-BMVC fluorescence, and their time-gated FLIM images with time threshold at 2.4 ns to separate the image into two colors: white (decay time ≥ 2.4 ns) and red (decay time < 2.4 ns) in live cells.

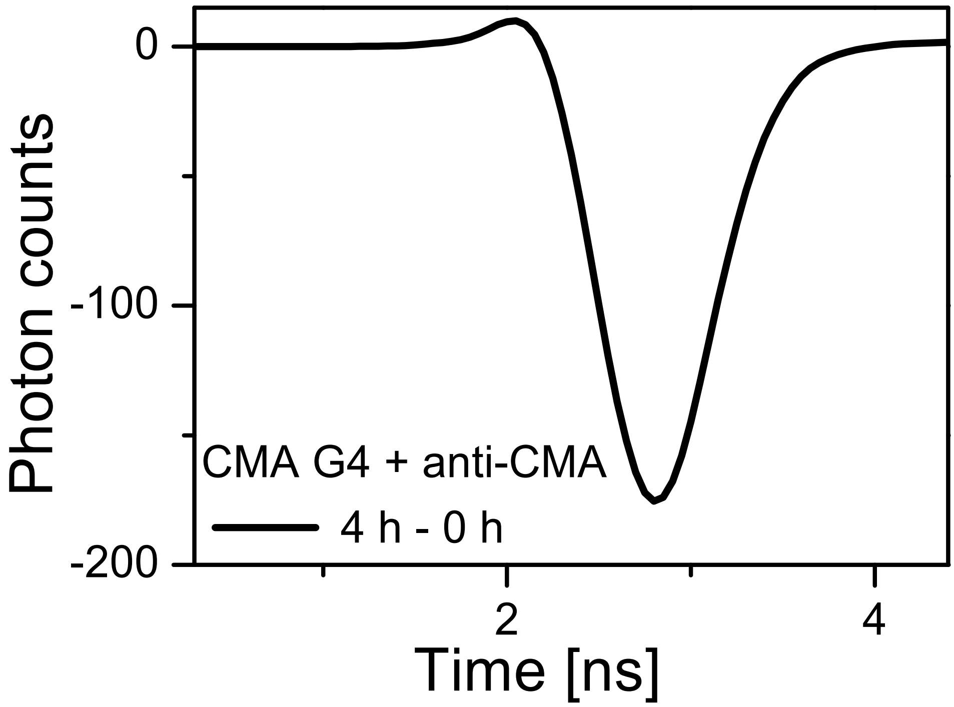

Figure S3. CD studies of CMA in vitro. The difference between the histograms of average photon counts of *o*-BMVC fluorescence per cell before and after the addition of anti-CMA at 4 h.
